# Unobtrusive ambulatory EEG using a smartphone and flexible printed electrodes around the ear

**DOI:** 10.1038/srep16743

**Published:** 2015-11-17

**Authors:** Stefan Debener, Reiner Emkes, Maarten De Vos, Martin Bleichner

**Affiliations:** 1 Neuropsychology Lab, Department of Psychology, European Medical School, University of Oldenburg, Oldenburg, Germany; 2 Cluster of Excellence Hearing4all, University of Oldenburg, Oldenburg, Germany; 3 Neurosensory Science, University of Oldenburg, Germany; 4 Institute of Biomedical Engineering, University of Oxford, Oxford, UK

## Abstract

This study presents first evidence that reliable EEG data can be recorded with a new cEEGrid electrode array, which consists of ten electrodes printed on flexible sheet and arranged in a c-shape to fit around the ear. Ten participants wore two cEEGrid systems for at least seven hours. Using a smartphone for stimulus delivery and signal acquisition, resting EEG and auditory oddball data were collected in the morning and in the afternoon six to seven hours apart. Analysis of resting EEG data confirmed well-known spectral differences between eyes open and eyes closed conditions. The ERP results confirmed the predicted condition effects with significantly larger P300 amplitudes for target compared to standard tones, and a high test-retest reliability of the P300 amplitude (r > = .74). Moreover, a linear classifier trained on data from the morning session revealed similar performance in classification accuracy for the morning and the afternoon sessions (both > 70%). These findings demonstrate the feasibility of concealed and comfortable brain activity acquisition over many hours.

Presently, the non-invasive monitoring of human brain activity is bound to stationary, highly controlled and often artificial laboratory conditions^1^. While research-quality electroencephalogram (EEG) systems can be made much smaller than other established brain activity recording technologies such as magnetic resonance imaging (MRI), near-infrared spectroscopy (NIRS) and magnetoencephalography (MEG), modern EEG systems still weigh several kilograms. Consequently traditional EEG has limited portability and data acquisition is cumbersome outside the laboratory. EEG acquisition in naturalistic environments has been identified as an important goal^2^,^3^. Here we present a new type of EEG electrode designed to enable unobtrusive and concealed EEG acquisition. We asked whether a portable and smartphone-operated, near-invisible EEG allows the collection of good-quality signals over a longer period of time.

Wired EEG systems limit natural behaviour of the participants during signal acquisition and thereby lead to heavily constrained recording conditions^2^,^3^. Recently established concepts such as natural cognition^2^ and active sensing^4^ strongly suggest that evidence obtained during highly controlled, artificial recording conditions may not capture very well the fundamental mechanisms of perception and cognition. The ambulatory assessment of brain activity may serve as a complementary approach to traditional laboratory studies, validating established theories under more naturalistic conditions. For this purpose miniaturized EEG mobile systems have been developed^5^,^6^. We found that a head-mounted wireless EEG system that was directly attached to an EEG electrode cap enables good portability and a solid degree of motion tolerance^1^,^7^. Good quality event-related potentials (ERPs) were obtained in seated and outdoor walking conditions and single-trial EEG analyses confirmed this conclusion. Wireless miniaturized EEG systems, which are designed to limit mechanical displacement of isolated parts of the measurement chain, can increase the degree of EEG portability and motion tolerance^8^.

A clear drawback of currently available mobile EEG systems is that they require additional hardware for signal acquisition and stimulus presentation, such as a notebook or computer. Modern high performance smartphones however are powerful enough to be used instead^9^ and indeed a few studies have reported successful EEG acquisition on a smartphone^6^,^10^,^11^. A second drawback of mobile EEG systems is that they rely on clearly visible EEG electrodes and clearly visible electrode caps or nets. This is a problem as it impedes people from recording EEG for longer periods of time in a convenient way. Alternatives to the standard EEG cap such as miniaturized wet sensors placed on the scalp^12^ as well as in and around the ear^5^,^13^,^14^,^15^ promise less visibility and better wearing comfort.

Aiming towards this goal we adopted the concept of flexible screen-printed electrodes^16^,^17^. The placement of disposable screen-printed electrodes in the face and forehead below the hairline has been shown to produce clinically useful information for the monitoring of altered mental states^18^. Based on our experience with miniaturized sintered Ag/AgCl sensors^13^, we designed a re-usable, flexible printed Ag/AgCl electrodes system consisting of ten electrodes arranged in a c-shape to fit around the ear (cEEGrid; Fig. 1). To allow for long-term recordings it is necessary that the electrode-skin impedance remains low and constant over time^19^. However, after an initial reduction of the electrode skin-impedance, which is facilitated by the filling of the ducts of the skin with electrolyte, impedance typically increases over time due to evaporation of the electrolyte solution^20^. Accordingly EEG signal quality often deteriorates the longer the electrodes are worn. The cEEGrid features miniaturized, wet sensors placed under a screen film, which acts like a seal. Despite the use of a minimum amount of gel, this design should limit electrolyte evaporation and therefore facilitate long-term recordings.

We tested whether good signal quality can be obtained over several hours with concealed, unobtrusive sensors. EEG was recorded wirelessly on a smartphone from ten individuals wearing a left and right cEEGrid from morning to late afternoon. Power changes in resting EEG data were analyzed to explore whether EEG recorded with the cEEGrid captures the well-known alpha power difference between eyes open and eyes closed conditions. In addition, using the same smartphone for concurrent stimulus presentation and EEG acquisition, auditory oddball data were recorded. ERP condition effects were analyzed and a single-trial analysis was conducted. Specifically, we tested whether a linear classifier trained on the single-trial EEG responses to target and standard tones presented in the morning would allow above chance-level classification of single-trial EEG data collected in the afternoon.

## Methods

### Participants

Twelve healthy volunteers free of past or present neurological or psychiatric conditions participated in the experiment. The sample consisted of undergraduate or graduate students or staff members of the Oldenburg Department of Psychology, most of whom had previous experience with EEG. Two individuals were excluded from the analysis. One was excluded due to technical problems resulting in loss of EEG signals, the other dataset had to be excluded because the participant did not follow the task instruction to count pitch-deviant tones. The final sample consisted of ten participants (23–47 years of age; mean 29.9 years; 5 male). Informed consent was obtained from all participants. Oldenburg University ethics committee approved the study protocol and all procedures were carried out in accordance with the approved protocol.

### Stimuli and Task

For the recording of resting EEG data participants were instructed to relax and keep their eyes open or closed, according to verbal instruction given by the experimenter. For the oddball task, two pure tone sounds (600, 900 Hz) of 62 ms duration (including 10 ms rise and fall time) were presented binaurally with consumer in-ear headphones (Samsung EHS64AVFWEG) at a participant-controlled, comfortable loudness. The 600 Hz tone was the standard tone, the 900 Hz tone was the target tone. Target probability was set to 20% and targets and standards were presented in randomized order, with the constraint that targets could not directly follow each other. A fixed inter-stimulus interval of 1000 ms was used and 860 stimuli presented on average per session. The task of the participants was to silently count the high, target tones while keeping the eyes open. To ensure that participants continuously paid attention the stimulus presentation was interrupted randomly between two and four times within a recording session and the participants had to report the number of silently counted target tones.

Stimulus presentation was performed with the Droid back-end of OpenSesame^21^ running on a Sony Xperia Z1 smartphone (model: C6903; OS: Android 4.4.4). The Android OS has limitations in supporting real-time demands. Initial timing tests revealed that audio presentation was not perfectly synchronized to the event markers sent into the EEG acquisition app. This caused a constant lag, which was moderately unpredictable across recording sessions but could be compensated for offline (see below). Despite rare temporal outliers occurring on less than 3% of all stimulus presentations, the temporal precision between marker time and stimulus presentation was found acceptable in pilot tests, when event markers were written after sound presentation (latency jitter approximately 6 ms standard deviation). This procedure was used and all markers were offline shifted back in time by a constant value (100 samples).

### Procedure

**Figure 1:**
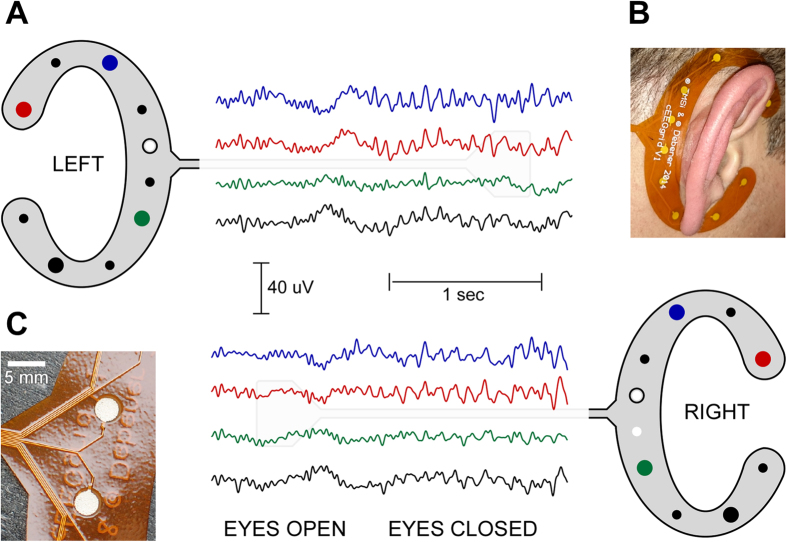
Illustration of the cEEGrid design and layout. (**A**) A few seconds of resting EEG recorded from a cEEGrid placed around the left and right ear. The two white electrodes illustrate positions of the reference channels (CMS top, DRL, bottom); off-line the data were re-referenced to algebraically-linked mastoids (white electrodes with black circle). (**B**) Picture of a cEEGrid placed around the right ear. A double-sided adhesive sticker with circular openings for the electrodes was used to attach the cEEGrid. Several sensors were placed at or above the hairline, while others were placed below the hairline. (**C**) Close-up view of two sensors and the wiring layout. Conductive surfaces are 3 mm in diameter and consist of dried, printed Ag/AgCl ink.

Participants arrived in the morning and were fitted with two cEEGrid devices (Fig. 1). This included preparation of the skin around the left and right ear using abrasive gel and alcohol swabs. Double-sided sticky tapes were attached to the cEEGrids, a drop of electrolyte gel (Abralyt HiCl, Easycap GmbH, Germany) was added onto the conductive surfaces, and then the cEEGrids were taped onto the skin around the ear. This preparation took less than five minutes on average. After the fitting of electrodes was completed the two cEEGrids were connected to a modified miniaturized amplifier (see below for details), which was placed with a headband at the back of the head. Impedance values in the range of 10 to 30 kΩ were available with a resolution of 5 kΩ. When impedance values were >30 kΩ, which occurred on average on less then 2 electrodes per subject, a blunted needle was used to add another drop of gel, without taking off the cEEGrid. Subsequently resting EEG data (2 × 1 min eyes open, 2 × 1 min eyes closed, in alternating order) and typical EEG artifacts (eye blinks, lateral eye movements) were recorded while the experimenter sitting next to the participant held the smartphone. For the resting EEG recording, event markers were added by touch screen presses executed by the experimenter, indicating different conditions (eyes open, closed, artifacts). After the resting EEG task was completed electrode impedances were recorded. Subsequently participants performed the auditory oddball task, which lasted approximately 20 minutes. Afterwards the amplifier was removed and the two cEEGrid connectors, one coming from the left ear and one from the right, were taped together to prevent them from dangling from the head. In the afternoon participants returned to the office and the cEEGrids were reconnected to the amplifier. The same recording protocol was repeated but in reversed order, that is, the oddball data were recorded first and the resting EEG second. Afterwards impedances were checked again. Note that between completion of the first recording session in the morning and the second in the late afternoon participants followed their normal daily activities, which consisted of office and laboratory work and in most cases included a lunch break, going for a walk and having a chat with friends or office mates. Participants were not monitored during this period and received no explicit instructions other then to avoid pulling on the cEEGrids when putting on glasses or headgear. In between the morning and the afternoon sessions no EEG was recorded and the experimenter did not manipulate the cEEGrid in any way. On average 6 hours and 14 minutes (min 6:00, max 7:21) passed between completion of the first oddball task in the morning and beginning of the second in the afternoon. Accordingly, the average period of wear of the cEEGrids was between 7 and 8:20 hours. All recordings were conducted in a seated position in a moderately quiet office environment. After the end of the afternoon session participants were encouraged to provide feedback on wearing the cEEGrids and were specifically encouraged to report potential discomfort. Finally the cEEGrids were removed and the participants received a tissue to remove residual conductive gel.

### EEG Recording

A SMARTING 24-channel mobile EEG amplifier was used (mBrainTrain, Belgrade, Serbia). The system features a sampling rate of 500 Hz, a resolution of 24 bits and a bandwidth from DC to 250 Hz (SMARTING, www.mbraintrain.com). The amplifier unit includes a 3D gyroscope and power supply for several hours use (weight 64 grams; size 82 × 51 × 14 mm), and transmits the data wirelessly with a bluetooth (v2.1) protocol to a paired device positioned nearby. The SMARTING amplifier was fitted with two secure digital (SD) memory card slots to connect to the two cEEGrids. The cEEGrids were designed as semi-disposable devices by the first author (S.D.) and Twente Medical Systems Inc. (TMSI, Oldenzaal, The Netherlands). The flexprint material included several layers of a biocompatible polymide. The conductive parts consisted of gold plated ends, pure copper traces, and conductive Ag/AgCl based polymer thick film ink. The conductive surface was circular with a diameter of 3 mm, and the distance between electrodes located within a cEEGrid was either 12 or 18 mm (center to center). The number of electrodes (10) as well as the size and shape of the cEEGrid version 1.0 as used in the present study were inspired by pilot recordings and previous experience with around the ear multi-channel EEG recordings using miniaturized sintered Ag/AgCl electrodes^13^. Signal acquisition was performed with an app running on Android and provided by the manufacturer of the amplifier (smarting version 1.0). Data from 18 EEG channels and event markers were written into a common file format (.bdf). Markers were generated by touch screen presses for the resting EEG recording and by OpenSesame for indicating sound presentation during the oddball paradigm.

### EEG Analysis

EEG data were analyzed off-line using EEGLAB version 13.4.4b^22^ and BCILAB version 1.1^23^ and custom scripts running under Matlab 7 (The Mathworks Inc., Natick, MA). The two electrodes in the middle of the right ear cEEGrid served as ground and reference. The left ear channel that did not have a homolog counterpart (i.e. the ground electrode of the right ear) was discarded and the remaining channels re-referenced to algebraically linked mastoids, resulting in a symmetrical 16-channel montage (8 channels per ear). For the resting EEG analysis data were high-pass filtered with a zero phase finite impulse response filter at 1 Hz (−6 dB cut-off at 0.5 Hz, filter order 1650) and for each condition, segments with a length of 1024 samples and an overlap of 256 samples were Hanning windowed, submitted to a fast Fourier transform (Pwelch method as implemented in Matlab), averaged and log-normalized (10*log10). Visual inspection revealed that a single channel (L1) from a single subjects’ afternoon EEG did not produce reliable signals, possible due to instable electrode-skin impedance. The corrupted spectrum at this single channel was replaced by the corresponding values from the identical channel recorded in the morning session. A similar problem did not occur for the analysis of the oddball data. Further analysis of the resting EEG results ensured that the bad channel replacement did not influence the results of the statistical evaluation. For the analysis of the oddball data, continuous data were finite impulse response filtered from 0.2 Hz (−6 dB cut-off at 0.1 Hz, filter order 8250) to 20 Hz (−6 dB cutoff at 22.5 Hz, order 331) and afterwards epochs from −200 to 800 ms were extracted and baseline corrected (−200 to 0 ms). Epochs dominated by artifact were identified using the probability and kurtosis criteria implemented in EEGLAB (standard deviation: 2) and rejected from further analysis.

To compensate for potential latency shifts caused by Android audio latency timing uncertainty a cross-correlation analysis between the target ERP global field power values of the morning and the afternoon oddball session was performed and lag differences were leveled by shifting all event markers by the lag identified. The average lag compensated for by this procedure was 15 ms (range across subjects: 0 to 46 ms). To further ensure that residual temporal uncertainty not accounted for by this procedure could not have an effect ERP latencies were not analyzed and the amplitude data analyzed were derived by averaging over large consecutive intervals of 100 ms width, starting at 0 ms. For the ERP analysis on average 79 target trials and 419 standard trials remained per session. The difference in the number of trials between morning and afternoon sessions was not significant (standards: t_9_ = 1.16, *p* = .28; targets: t_9_ = 1.67, *p* = .13).

Single-trial EEG analysis was performed using regularized linear discriminant analysis (LDA) classification as implemented in BCILAB. The continuous data were filtered from 0.1 to 6 Hz^24^. Epochs were extracted from 0 to 800 ms and epochs with artifacts (identified as described above) were discarded. The number of trials for each class (target, standard) was balanced, resulting in a chance level of 50%. The feature space used comprised five non-consecutive 100 ms mean windows (from 200 to 700 ms) and 16 channels, resulting in 80 features. To reduce the risk of over-fitting shrinkage LDA with default settings as implemented in BCILAB was used. Specifically a linear classifier was trained on the morning data and evaluated on the afternoon data. To obtain a valid performance estimate for the calibration data, a 5-fold chronological cross-validation resampling procedure was performed. The percentage of correctly classified trials (% accuracy) was taken as performance measure. Subsequently the influence of the number of channels on classification accuracy was explored with an iterative procedure^25^. To this end channels were ranked in descending order by calculating the discriminative value of each electrode by means of point bi-serial correlations. For each of the 16 iterations the least discriminative channel was discarded, classifier training on the morning data was repeated and the resulting model was evaluated on the afternoon data. Since channel selection was performed on the morning data and model evaluation was done on the afternoon data a circularity problem was avoided^26^.

### Statistical Analysis

To statistically test the difference between EEG power obtained from eyes open and eyes closed conditions EEG frequency bands were defined (1–3 Hz, 4–7 Hz, 8–12 Hz, 13–30 Hz, 31–80 Hz) and log band power values submitted to paired t-tests. As this resulted in a large number of statistical tests (16 electrodes ×5 frequency bands) a Bonferroni correction was applied (.05/80) and only effects below p = .000625 were considered significant. Oddball ERP analysis was performed with a 2 × 2 repeated measurements analysis of variance (ANOVA), comprising the factors Session (morning, afternoon) and Condition (target, standard). In order to explore whether the ERP data obtained with the cEEGrid contained meaningful spatial and temporal signatures the same ANOVA model was repeatedly applied to all channels and time bins used for the single-trial classification. Again a Bonferroni correction for multiple comparisons was applied (16 channels ×5 time bins; p = .000625). Further statistical evaluation included a test-retest reliability analysis for target ERPs by means of Pearson correlations and target versus standard ERP condition effect sizes were calculated by dividing the mean differences by the pooled standard deviation (Cohen’s d). To test whether classification accuracies were above chance-level a binomial statistic with a confidence limit of *p* = 0.05 was used^27^ and to evaluate whether a significant loss in classification accuracy occurred from morning to afternoon a paired t-test was applied.

## Results

All ten participants completed the study. No one gave an account of a painful experience by wearing the cEEGrids, but one reported being relieved when the grids were taken off after the end of the afternoon session. Three participants reported minor discomfort, three others reported that they were aware of the cEEGrids during the day but were not disturbed by it, and three reported that they forgot about wearing the cEEGrids most of the time. For individuals reporting discomfort visual inspection revealed that the edge of the cEEGrid had direct contact with the posterior auricle (see Fig. 1b, for an example). Apparently the shape and/or size of the cEEGrid did not perfectly fit to everyone’s ear anatomy.

Evaluation of signal quality by means of electrode-skin impedance measurement revealed an average impedance of 16.47 kΩ (standard deviation: 6.16 kΩ) in the morning. The group and channel average impedance in the afternoon was 16.31 kΩ (standard deviation: 5.97 kΩ), and this difference was not statistically significant, t(9) = 0.15, p = .89. Since these values reflect an average measure across all channels per subject, the same analysis was repeated for each individual channel. For none of the 16 channels a significant change in impedance was found (all t > = 1.47, all p > = .177; mean t = 0.71; standard deviation: 0.52).

**Figure 2:**
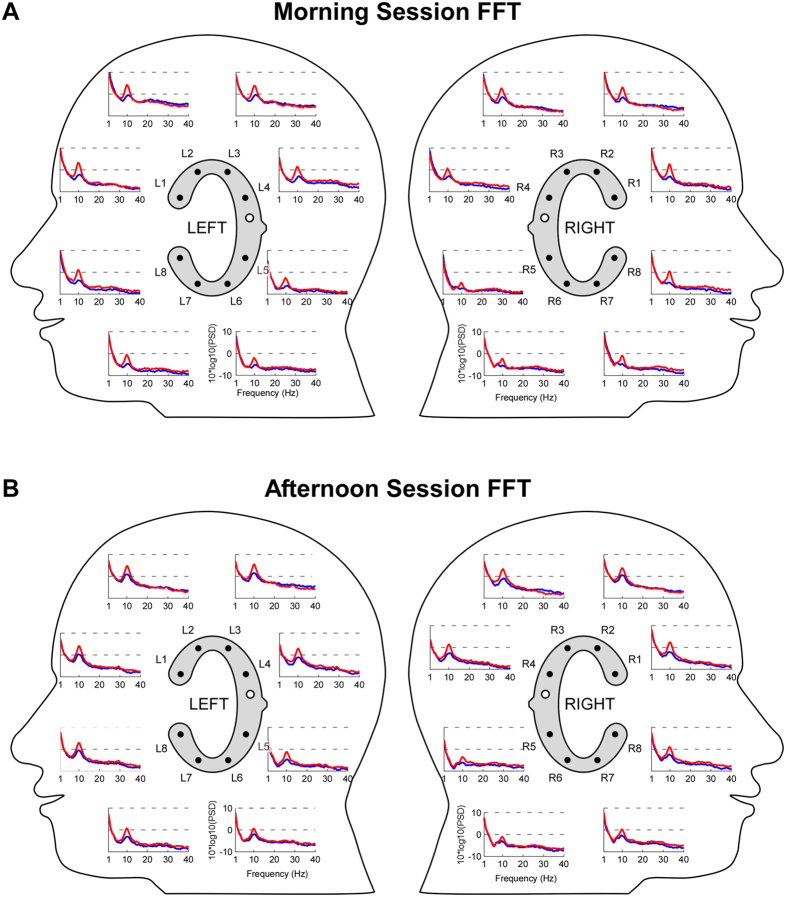
Grand average (N = 10) EEG spectra as recorded from left and right ear cEEGrids, shown in a 2D lateral view layout. Electrode labels are indicated. Blue: eyes open; red: eyes closed; FFT: fast Fourier transform. White electrodes with black circle in inset indicate the off-line reference position. (**A**) Grand average EEG spectra from the morning session. (**B**) Grand average EEG spectra from the afternoon session.

**Figure 3:**
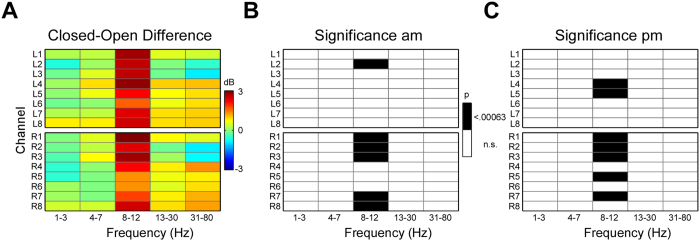
Spatio-temporal cEEGrid images illustrating EEG results as a 16 channels × 5 frequency bands matrix. Frequency bands reflect the mean power for the frequency ranges indicated; electrode labels indicate positions as illustrated in Fig. 2. (**A**) Image representation of the EEG power difference between eyes closed and eyes open, in dB. (**B**) Eyes open versus eyes closed condition effect for the morning session, with significant effects (<.000625) shown in black. (**C**) Same as B for the afternoon session.

Only 0.3% of all recorded channels were unreliable in the afternoon. Specifically a single channel (L1) from a single afternoon dataset did not contain signals resembling typical EEG in the resting EEG condition, resulting in a bad channel replacement. Nine out of ten participants showed a clear increase in EEG 8–12 Hz alpha power for eye closed compared to eyes open. Figure 2 illustrates the spatial pattern for this effect, separately for the morning and afternoon recordings. A clear peak in the alpha frequency range was evident in most channels and alpha power clearly varied in magnitude across channels. The spatial profile of the difference in EEG power between eyes open and eyes closed is illustrated for all frequency bands in Fig. 3 (left). As can be seen, the condition difference in log power was strong in the 8–12 Hz alpha band (>2.5 dB for eight channels), small (<1.5 dB for four channels) in the gamma frequency band and absent in all other bands (<1 dB). Figure 3 shows also the results of the statistical evaluation of the condition difference, separately for the morning (center) and the afternoon session (right). Significant effects (p < .000625) were found only for the 8–12 Hz alpha frequency band and were evident for six channels in the morning and seven channels in the afternoon. For four of these channels the result was robust over time.

**Figure 4:**
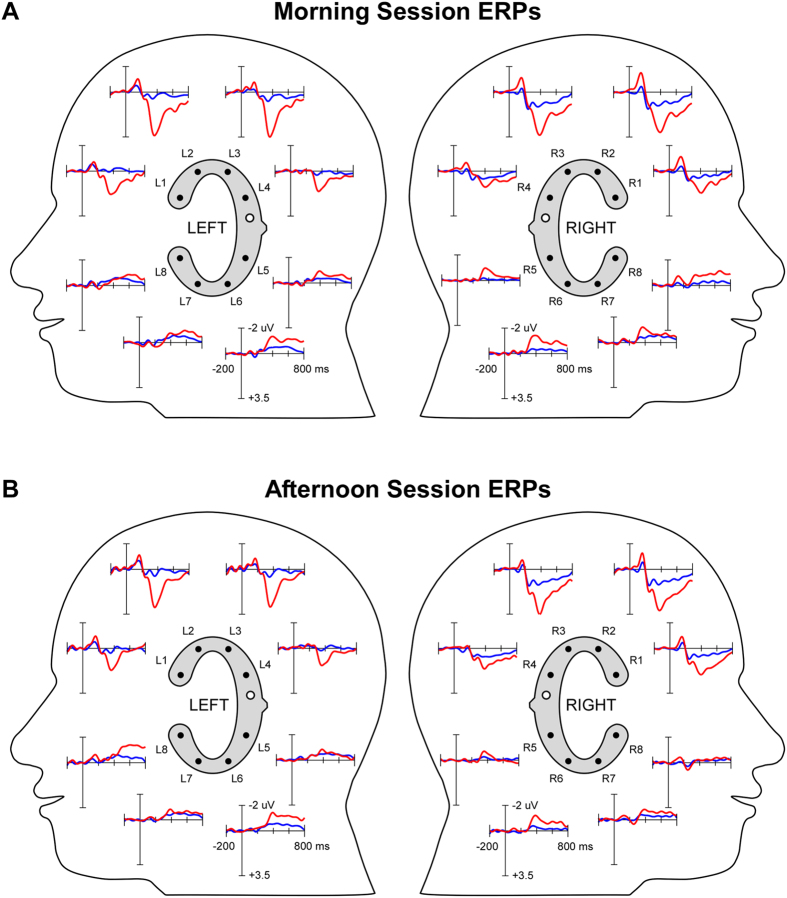
Grand average (N = 10) ERPs as recorded from left and right ear cEEGrids, shown in a 2D lateral view layout. Electrode labels are indicated. Negative voltage is plotted upwards. Blue: standard condition; red: target condition. White electrodes with black circle in inset indicate the off-line reference position. (**A**) Grand averaged ERPs from the morning session. (**B**) Grand average ERPs from the afternoon session.

ERPs for target and standard tones are illustrated in Fig. 4. Both tones gave rise to a negative deflection approximately 100 ms after tone onset, resembling an auditory evoked potential N100 component e.g.^28^. This deflection was pronounced on channels L1 to L3 on the left side and R 1 to R3 on the right and was diminished or absent on the other channels. Channels R7 and R8 showed a polarity-reversed pattern of an otherwise similar morphology. This pattern was not (or to a much lesser extend) visible at the homolog channels from the left ear. More prominent than the N100 was a positive deflection in response to target tones, with maximum amplitudes at approximately 400 ms. Morphology, condition effect and latency of this deflection strongly resembled the typical P300 ERP component cf.^29^. In a similar manner to the N100, the P300 was pronounced for channels located above the reference sites (shown in white in Fig. 4), that is, channels L1 to L4 and R1 to R4. Below the reference an opposite polarity waveform emerged, which was most pronounced at channels L6 and R6. Visual inspection of the ERP results for the morning (Fig. 4 top) and afternoon (bottom) session reveled overall similar waveform morphologies, amplitudes and condition effects.

**Figure 5:**
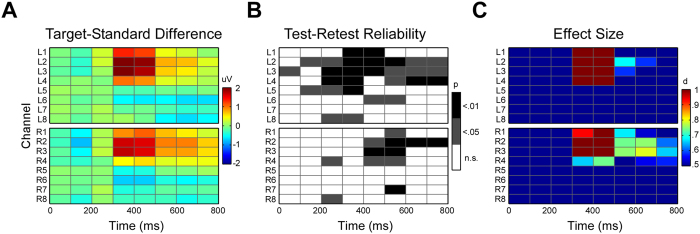
Spatio-temporal cEEGrid images illustrating ERP results as a 16 channels × 8 time bins matrix. Time bins reflect the mean signal over 100 ms bins, electrode labels indicate positions as illustrated in Fig. 2. (**A**) Image representation of the ERP voltage difference between targets and standards. (**B**) Test retest reliability results, with significant correlations shown in darky grey (r > = .602, p < .05) and black (r > = .735, p < .01). (**C**) Effect size results (Cohen’s d). Large effects (d > .5) are indicated.

Parameterization of the ERPs involved calculation of time window means for each channel and eight consecutive 100 ms bins, resulting in a 16 (channels) times eight (time bins) matrix. Figure 5 (left) shows the amplitude difference between ERPs for target and standard conditions, averaged over morning and afternoon recording sessions. As also indicated by Fig. 4, the condition effect was most pronounced between 300 and 500 ms and maximal at channels L2 and 3 over the left hemisphere and homolog channels R2 and R3 over the right. In order to quantify the test-retest reliability of the condition effect Pearson correlations were calculated. This was done for the target to standard ERP difference for all channels and time bins, and the results are illustrated in Fig. 5 (middle), with thresholds set for correlations r > = .62 (p < .05) and r > = .735 (p < .01). Forty out of the 128 variables tested were significant on a p < .05 level, among them 19 on a p < .01 level. The pattern of significant time bins corresponded partly with the topography of the condition effect (Fig. 5 left), with pronounced re-test reliability effects on many channels in the time range from 300 to 600 ms, with a preference for more reliable effects over the left (28) compared to the right (12) cEEGrid. To further characterize the spatio-temporal topography of the target to standard condition effects an effect size measure (Cohen’s d) was calculated for each channel and time window (Fig. 5 right), after averaging over the morning and the afternoon ERP data. Very large condition effects (d > .9) were evident for channels L1 to L3 and R 1 to R3 in the 300 to 500 ms latency range, overall showing good resemblance to the amplitude difference topography (Fig. 5 left).

The Session (morning, afternoon) by Condition (target, standard) 2-way repeated measurements ANOVA was repeatedly conducted for the 100 ms time bins from 200 to 700 ms and each electrode as dependent variable. This was done to test whether the predicted significant ERP condition effect could be verified statistically and whether a possible interaction with Session would emerge. For none of the 80 ANOVA models calculated was a main effect of Session found, and the same was evident for the Session by Condition interaction (all p > .00625). Only the main effect of Condition remained significant after Bonferroni correction. For the 300 to 400 ms time bin the Condition effect was significant for 6 of the 16 electrodes, with values between F(1,9) = 28.52, p = .00047 and F(1,9) = 179.25, p = .0000003.

**Figure 6:**
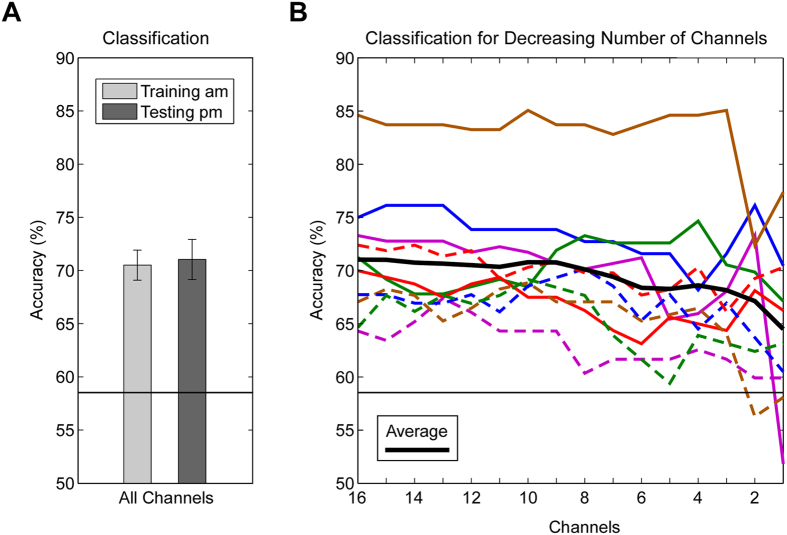
EEG single-trial classification results plotted as percent accuracy correct. A black horizontal line indicates the statistical chance-level. (**A**) Group mean classification results for the morning training data (am) and the afternoon testing data (pm) when 16 channels were used for classifier training and testing. Error bars indicate ± one standard error of the mean. (**B**) Single-subject (colored lines) and group average (bold black line) classification results for decreasing number of channels, from 16 to one. Channel selection and classifier training was performed on the morning session data, shown are the evaluation results for the afternoon session data.

In order to evaluate the single-trial EEG quality a classification analysis was conducted. For each subject a linear classifier (shrinkage-LDA) was trained on the difference between target and standard trials using 80 features (16 electrodes, 5 consecutive time bins from 200 to 700 ms). Classifier training was limited to the morning data and a 5-fold cross-validation allowed for the interpretation of classifier performance for the morning data. Subsequently this classifier was evaluated on the afternoon data. As shown in Fig. 6 (left) the classifier accuracy was clearly above chance-level. The average accuracy was 70.34% in the morning (range: 64.75–79.0%) and 70.92% in the afternoon (range: 64.32–84.62%). No significant difference in classification performance emerged between morning and afternoon, t(9) = −0.31, p = .77. The classification results were also above chance-level for all subjects individually (not shown).

Adopting an iterative channel selection procedure cf.^25^ we also evaluated how strongly the classification performance depended on the number of channels used for training. On each iteration step the least discriminative channel was removed and the resulting classifier was evaluated on the afternoon data. As can be seen in Fig. 6 (right), on a group average level a modest reduction in classification performance from 71% with 16 channels to 65% with a single channel was found. Evaluation of the single subject results revealed individual differences. While some individuals optimal classification results were only achieved using all channels, others showed very similar, if not better classification performance for fewer channels. Interestingly, the best participant showed robust performance between 84 and 85% classification accuracy from 16 channels down to 3 channels, and only a further channel reduction led to a strong decline in classification accuracy.

## Discussion

To acquire brain activity in naturalistic settings the recording setup should be as unobtrusive as possible and hidden from view to other people. To achieve this we combined a lightweight mobile EEG amplifier with a smartphone for signal recordings and developed a concealed behind-the-ear electrode array. Well-known EEG oscillations and ERP patterns, such the eyes closed alpha effect and the P300 component elicited in response to task-relevant events, could be recorded with this setup. The cEEGrid, which is a wearable flexible printed electrode array, enables the acquisition of good quality multi-channel EEG signals over many hours, without significant user discomfort or distraction from daily life routines. In combination with the miniaturized EEG amplifier and an off-the-shelf smartphone used for stimulus presentation and signal acquisition the setup used in this study fits into a trouser pocket, a desirable feature for various different EEG applications.

In a recent study^13^ we complemented and replicated previous research by showing that miniaturized EEG electrodes are sufficient for the collection of good quality ERPs^12^. This study showed that electrodes located in and around the ear allowed the recording of ERPs in a standard brain computer interface (BCI) paradigm. Specifically, despite overall smaller amplitudes, we found a comparable P300 condition effect size for an above the ear channel (approximately 10–20 position T8, referenced to an electrode in the concha) when compared to a traditional scalp EEG channel (10–20 scalp position Pz, referenced to mastoid). While a shorter distance between two electrodes forming a (bipolar) channel increases the common mode and thereby reduces measured amplitudes, the same effect holds for undesired influences, such as far-field noise, resulting in comparable signal-to-noise levels for large distance and small distance channels.

The present data fit well to this previous interpretation of rich spatio-temporal detail captured by in an around the ear EEG. Here we report typical oddball ERP condition effects as have been reported by numerous scalp EEG studies^29^. Accordingly, we provide further evidence for the value of behind and around the ear EEG^5^,^13^,^14^,^15^. Compared to the setup used by these authors, the cEEGrid array the clear advantage of providing a better (and well defined) spatial sampling, which facilitates the identification and separation of overlapping brain signals and artifacts^30^. The present data show a clear spatial pattern for the P300 reminiscent of the spatial P300 pattern we reported before^13^. However, future concurrent high-density EEG and cEEGrid studies are needed to exactly quantify the amount of spatial information that is present in cEEGrid recordings in comparison to traditional scalp EEG. We expect that, for an optimal orientation of the recording electrodes relative to the electrical moment of a dipolar generator, the loss in information may be negligible. This prediction could be best tested by comparing cEEGrid and scalp EEG effects for a number of experimental paradigms known to draw on cortical generators from different locations. In addition, such a study design would allow a comparison of the wearing comfort of the cEEGrid and the traditional EEG cap.

We designed the cEEGrid for good quality long-term signal acquisition, repeated use, good user comfort and low visibility. Practical experience with the cEEGrid confirmed that it is very convenient to use. Skin preparation and array application time as well as array removal and cleaning are marginal compared to scalp EEG. The visibility (ranging from visible to completely concealed) depended on the participant’s hair-style (cf. Fig. 1). Despite these desirable features further improvements could be implemented. The electrode application time could be further reduced by the use of a hydrogel membrane directly attached to the electrode^16^,^17^,^18^,^20^,^31^, which would also help to further stabilize impedances over longer periods of time. A very fast application time could make the cEEGrid also interesting in the context of emergency EEG. Future versions could also reduce visibility further by use of translucent material, a smaller adherent, smaller electrodes and variable or individualized array sizes and shapes. The latter aspect would be helpful in order to achieve better wearing comfort in individuals with unusual ear anatomy. Regarding repeated use, several of the datasets reported in the present study were acquired with previously used grids. Modifications in the design could be easily tailored towards the production of either very cheap, disposable arrays or more sturdy arrays that could be used repeatedly.

To achieve a high level of portability we used a small amplifier in combination with an off-the-shelf smartphone running an Android OS for signal acquisition and sound presentation. Though this setup was functional in general and is very promising for further experiments, some limitations nevertheless apply. The Android OS as used in this study (Android OS 4.4) is not designed for supporting real-time applications. This provides a significant limitation for the acquisition of time-resolved ERPs. In the current study, we used 100 ms bins for the statistical analysis and an offline correction procedure to synchronize ERPs. For future mobile EEG systems better, real-time capable operating systems for smartphones are desirable.

The very good ERP amplitude reliability along with the excellent ERP condition effect size supports the notion that good quality ERP data were recorded with the cEEGrid electrodes array. Importantly, skin-electrode impedances did not increase over the course of six to seven hours, as could be expected by the design of “sealed” electrodes. We are not aware of any previous study reporting long-term stable electrode skin impedances using miniaturized electrodes and a conventional electrolyte gel. Adhesive electrode paste such as EC2 is routinely used in 24 h epilepsy monitoring and enables stable impedances^32^, but usually this setup comes with head bandages and poor user comfort. Use of EC2 gel with the cEEGrid may help to prolong stable impedances and thus increase recording durations even further. The development of novel hydrogels as reported by Kleffner-Canucci^20^ is another interesting option towards convenient, long-term EEG monitoring.

The ERP amplitude quality is also documented by the single-trial EEG analysis performed. A linear classifier (LDA) trained on the morning data was found to produce very similar classification results for morning data (obtained via cross-fold validation) and unseen afternoon data. Since the model performed without additional loss on the unseen data, overfitting was apparently not an issue. The overall moderate classification accuracy is probably inherent in the oddball design used. Previous single-trial oddball classification studies reported similar, or only moderately better classification accuracies with scalp EEG data^1^,^33^. More important than overall classifier accuracy is the stability of the classifier performance. The generalizability of classifiers is a general problem for BCI systems. To overcome EEG signal non-stationarities (e.g. due to fatigue, change in task involvement or change in recording quality) classifiers are generally adapted from session to session^34^,^35^. The stable classification achieved here strongly suggests that the signal was fairly stationary over the two sessions. A possible explanation is the tight contact between electrode and skin avoiding non-stationarities that are otherwise due to electrode displacement and electrode-skin impedance changes, as they inevitably happen with a normal EEG cap. Individualization, for instance regarding the number of electrodes necessary for a particular application, may also be a further way to improve the practicability of EEG acquisition in daily-life settings. Finally, for a further development and validation of mobile EEG^7^ the motion-tolerance of the cEEGrid array has to be tested.

In summary, we present a new approach for unobtrusive and concealed EEG acquisition. Printed flexible screen technology enables the low cost production of extremely lightweight and comfortable electrode arrays and thereby contributes fundamentally to the development of wearble electroencephalography^36^. We envision different applications for this technology, including EEG sensing for hearing devices, studies of social interaction, pediatric EEG, long-term EEG epilepsy monitoring, sleep EEG and BCI application in daily-life settings. In the present case the electrodes were arranged around the ear but other configurations may be interesting as well. In any case, the good signal quality found with the cEEGrid suggests that this new approach could help to pave the way towards robust, daily-life EEG recording.

## Additional Information

**How to cite this article**: Debener, S. *et al*. Unobtrusive ambulatory EEG using a smartphone and flexible printed electrodes around the ear. *Sci. Rep*. **5**, 16743; doi: 10.1038/srep16743 (2015).
